# Research on the underlying dynamics of leisure’s impact in arts venues

**DOI:** 10.3389/fpsyg.2025.1401922

**Published:** 2025-06-18

**Authors:** Hanbing Yang

**Affiliations:** Chelsea College of Art and Design, University of the Arts London, London, United Kingdom

**Keywords:** SOR framework, emotional factor, mediation effects, art venues, qualitative research and analysis

## Abstract

Amid the worldwide rise in cultural and creative tourism, art spaces are drawing increasing attention for their engaging and restorative properties. This research integrates both conceptual literature and empirical findings—examining the subject from multiple angles—to illuminate how public art environments affect visitors’ emotions and level of involvement. Although extensive work has explored art’s effects on emotions and psychology, the specific impact of emotional factors on visitor experiences within these venues remains under-investigated. Thus, this paper employs the SOR framework alongside mediation theory to delve into the connection between visual cues and visitor satisfaction in art settings. Data collected from 603 participants were analyzed using structural equation modeling, which indicated that aesthetic experience mediates the link between visual quality and satisfaction, while cultural identity moderates the relationship between visual quality and aesthetic experience. These findings aim to equip managers with deeper insights into how visual attributes and emotional responses interact, thereby offering practical strategies for enhancing satisfaction in creative art tourism destinations.

## 1 Introduction

Emotional experience is considered a key factor in landscape tourism research, influenced by the physical attributes of the landscape and exerting profound effects on overall engagement and satisfaction (Hosany and Gilbert, 2010; He et al., 2019; Hu et al., 2021). Art spaces, as important carriers of urban culture and art, offering unique appeal and experiential value to visitors, the process of enriching visitors’ visual experiences is often influenced by emotional factors (Yan et al., 2019; Tang et al., 2020). In a typical ploy, in 2014, the imitation Sphinx and mishmash pagoda in Hebei Province, China, generated negative emotions and discontent among visitors, leading to social controversy and ultimately facing the fate of demolition (Yin and Qian, 2020). It follows then that not only is art creation is influenced by the emotions of its creators, but people can also gain new insights and experiences through artworks. According to statistics on the number of publications in the field of art therapy over the past decade (2012–2022) from Web of Science, research focusing on art therapy has widely explored the role of artworks in emotional regulation and psychological disorders (Harris et al., 2018; Hu et al., 2021). However, fewer studies have extended the range of affective changes induced by artistic situations to predict tourist responses and behavioral decisions. Considering tourists as one of the main consumers of urban public space, understanding the emotional experience of tourists and their mediating role in the art tourism experience is particularly important for the overall satisfaction of the destination and marketing decisions (Prayag et al., 2013; Bonnefoy-Claudet and Ghantous, 2013).

According to data published by China’s Ministry of Culture and Tourism, the milestone of a per capita GDP exceeding USD 5,000 in 2011 signified a paradigm shift in domestic tourism preferences—from conventional travel motivations toward the pursuit of more spiritually enriching experiences (Ma and Zhou, 2019). In parallel, national art museums in China enjoyed a sustained increase in visitor numbers until the COVID-19 pandemic, when enforced travel limitations led to a transient decline. The continuous rise in per capita income, together with the escalating allure of art venues, suggests that these cultural institutions successfully deliver emotionally gratifying experiences—such as enjoyment and relaxation—that meet deeper spiritual aspirations and thus stimulate a significant influx of visitors.

Given this empirical and theoretical context, it becomes evident that emotional responses function as a pivotal conduit linking the visual stimuli offered by art venues to subsequent visitor behaviors. This underscores the importance of thoroughly investigating the factors that shape these affective responses. Anchored in the stimuli–organism–response (SOR) framework, the present study scrutinizes how the visual appeal of art spaces influences tourist satisfaction by assessing the mediating role of emotional factors within the creative tourism experience. The objective is to generate insights that can inform strategic planning and management practices for urban art institutions.

The contributions of this research can be delineated into three primary aspects:

1. Elucidation of Affective Mediation: The study rigorously examines how emotional experiences underpin visitor satisfaction, thereby clarifying the intermediary role of affect in shaping responses to art environments.
2. Development of an Integrated Theoretical Framework: By synthesizing the SOR model with mediation effect theories from modern cognitive psychology, this research offers a robust theoretical basis for understanding how emotional factors mediate the relationship between the visual quality of art venues and tourist behavior.
3. Practical Strategic Recommendations: Based on empirical mediation analysis, the study advances targeted suggestions aimed at enhancing the tourist experience at art attractions and refining urban spatial management.

The structure of this paper is as follows: Section 2 provides an extensive literature review, discussing both the evolution of research on emotional experiences in tourism and the application of the SOR model. Section 3 presents the theoretical framework and formulates a series of hypotheses regarding the influence of art venue aesthetics on visitor experience and behavior. Section 4 outlines the methodology, including the design of measurement instruments and the data collection process. Section 5 details the results of the data analysis, incorporating exploratory factor analysis (EFA) and confirmatory factor analysis (CFA) to evaluate the proposed hypotheses. Section 6 offers a discussion of the findings and articulates the study’s conclusions, while Section 7 addresses the limitations of the research and proposes directions for future inquiry.

## 2 Literature review

This research investigates visitor experiences within China’s art districts by examining the interplay between visual quality, emotional responses, and overall satisfaction. The objective is to uncover the psychological mechanisms underlying how individuals engage with public art spaces. To this end, we integrate the stimuli–organism–response (SOR) framework with mediation effect theory, formulating a series of hypotheses regarding the influence of visual characteristics on tourist satisfaction and the intermediary role played by emotional experiences. The resulting model offers specific recommendations for the planning and management of public art spaces. This study investigates visitor experiences in public art spaces by integrating three key research areas: landscape evaluation and satisfaction, emotional experience in tourism, and the SOR model with mediating effects. It examines how visual quality influences visitor satisfaction through emotional responses, emphasizing the mediating role of affective experiences. The study employs SWOT analysis and Importance-Performance Analysis (IPA) to assess visitor perceptions, focusing on how individuals interpret and emotionally engage with public art. It explores attitude variables, including aesthetic cognition, prior experiences, and cultural background, which shape visitor evaluations. Additionally, it analyzes affective experiences, identifying how emotions such as pleasure, nostalgia, relaxation, and curiosity emerge in public art spaces. These emotional reactions contribute to outcome variables, such as visitor satisfaction, intention to revisit, and loyalty to art spaces. The study applies the SOR model, linking external stimuli (visual elements of public art) to internal emotional processing and behavioral responses. Furthermore, it investigates the mediating effect of emotions, illustrating how affective experiences serve as a bridge between visual perception and visitor satisfaction. By addressing the research gap in the psychological mechanisms of visitor engagement, this study provides empirical insights and practical recommendations for optimizing the planning, management, and experiential design of public art spaces.

### 2.1 Research on landscape assessment and visitor contentment

Given the significant role of landscapes in shaping destination appeal and enhancing its overall image, the evaluation of landscapes and corresponding satisfaction levels have emerged as crucial topics in urban planning and tourism studies (Moon and Han, 2019; Fornal-Pieniak and Żarska, 2022). A review of existing literature on landscape assessment and satisfaction reveals four key dimensions: research focus, theoretical foundation, study content, and methodological approach.

From the research focus perspective, studies generally investigate specific landscape elements (Liu et al., 2023), individual landscape types (Fornal-Pieniak and Żarska, 2022), or comprehensive landscape environments (Huu et al., 2018) to identify primary factors influencing landscape perception and develop targeted optimization strategies. The scope of landscape assessment has evolved from analyzing isolated aesthetic attributes to examining entire landscapes. However, most research predominantly focuses on natural environments such as forests (Liu et al., 2023), wetlands (Das and Basu, 2020), and mountainous regions (Ding et al., 2022), with limited attention given to public art spaces and art venues. As art spaces play an increasingly significant role in urban renewal and cultural tourism development in China, this gap in research warrants further exploration.

In terms of theoretical foundations, landscape evaluation studies adopt interdisciplinary perspectives to assess how different landscape dimensions impact human preferences and satisfaction. These studies reference theories such as landscape performance theory (Xiang et al., 2022), cultural ecosystem service frameworks (Huu et al., 2018; Mao et al., 2020), planned behavior theory (Tabatabaie et al., 2023), and the American Customer Satisfaction Index Model (Xie et al., 2023). Despite incorporating diverse theoretical frameworks, research rooted in modern psychological theories remains scarce. Studies that do apply psychological perspectives often focus on visitor experiences but neglect behavioral responses and the mechanisms linking landscape characteristics with visitor engagement (Liu et al., 2023). A notable development in recent research is the increasing recognition of user evaluations, particularly in urban green space studies (Mao et al., 2020; Chen et al., 2019; Keith and Boley, 2019), signaling a shift toward socially practical and sustainable approaches to landscape assessment. Some research now prioritizes urban residents’ and tourists’ preferences and satisfaction, aiming to generate empirical data that informs landscape resource management and sustainable tourism planning (Mao et al., 2020; Zhang et al., 2022).

From a methodological standpoint, landscape assessment studies commonly utilize analytical frameworks such as SWOT analysis (Kazemi et al., 2018), Importance-Performance Analysis (IPA) (Das and Basu, 2020; Keith and Boley, 2019), and behavioral and scenario-based planning models (Weber et al., 2019) to capture resident and visitor perceptions. These approaches provide a scientific basis for future landscape planning and tourism management, offering actionable insights to guide decision-making and optimize landscape resource utilization.

Recent studies have increasingly emphasized how individuals perceive and interact with different landscape environments. Table 1 summarizes key findings from research exploring public opinions, particularly how city residents evaluate their surroundings (Das and Basu, 2020; Mao et al., 2020) and how travelers engage with diverse spatial settings (Ding et al., 2022; Zhang et al., 2022). While these studies contribute to a broader understanding of environmental assessment, many rely on structured classification methods and predefined models, limiting a more comprehensive analysis of human-environment interactions. Although landscape research has made significant progress, there remains a gap in understanding the psychological mechanisms that shape visitor experiences, particularly in artistic environments. Since cultural and artistic spaces enhance urban aesthetics and serve as major tourist attractions, further investigation is needed to explore how these settings influence visitor satisfaction. Studying the relationship between artistic composition, spatial arrangement, and visitor engagement can provide valuable insights for improving urban planning and cultural site development.

**Table 1:** Representative research literature on landscape evaluation and satisfaction (2018–2023).

Author	Published time	Research object	Research method	Research contribution
Liu et al. (2023)	2023	Forest landscape	Attention recovery theory	Analyze how variations in color across different forest environments influence psychological well-being, and propose design strategies to enhance landscape aesthetics and mental health benefits.
Fornal-Pieniak and Żarska (2022)	2022	Vegetation	Landscape natural resources evaluation	Analyze the influence of vegetation on tourism appeal in Central Europe, offering a scientific foundation for optimizing the development of natural tourism destinations.
Das and Basu (2020)	2020	Wetland	IPA	Investigating how various ecosystem services influence satisfaction levels and highlighting the significance of wetlands for both urban populations and nearby rural communities.
Xiang et al. (2022)	2022	Urban park	American landscape performance system	Analyzing environmental, health, and economic performance to identify suitable strategies for optimization and enhancement.
Mao et al. (2020)	2020	Green spaces	Cultural ecological service systems	Assess the satisfaction levels of residents with urban green spaces and propose recommendations for their planning and design improvements.
Huu et al. (2018)	2018	Street	Cultural ecological service systems	Analyzing the cultural value of seven major tourist destinations in Vietnam and emphasizing the significant relationship between landscape-derived cultural benefits and individuals’ well-being.
Kazemi et al. (2018)	2018	Shrub and arboriculture	SWOT	Develop a set of strategic recommendations tailored to support the growth of urban agriculture in developing nations.
Weber et al. (2019)	2019	Climate change	Scenario planning and landscape visualize	Investigate how climate change influences visitor satisfaction and identify strategies to balance ecological conservation in parks with visitor expectations.
Tabatabaie et al. (2023)	2023	Street (sidewalk and greening element)	Theory of planned behavior	Investigate the connection between perceived streetscape features and walking patterns among individuals in underserved communities.
Xie et al. (2023)	2023	Work environment (park, green space)	American customer satisfaction index model	Develop a conceptual framework to examine how the work environment influences entertainment satisfaction, offering insights and recommendations for enhancing the quality of green space services.
Chen et al. (2019)	2019	Green space	Eight sensory dimensions	Analyzed the effectiveness of eight sensory dimensions in public perception and identified their correlations with individual preferences.
Keith and Boley (2019)	2019	Greenway	IPA	Emphasize user satisfaction while addressing the research gap in understanding differences in perceptions and preferences for greenways between residential and non-residential users.
Zhang et al. (2022)	2022	Forest	Eye tracking	By identifying variations in how different spatial perception factors influence tourists’ visual behavior, this study provides practical recommendations for optimizing the design and development of high-quality forest parks.
Ding et al., (2022)	2022	Mountain	IPA	The perceptual experience is primarily driven by visual perception, with smell playing a secondary role, while touch, hearing, and taste have minimal influence.

### 2.2 Integration of emotional experience in tourism studies

Emotions have gradually become a central theme in tourism research, expanding beyond consumer behavior studies into a broad, multidisciplinary context. This shift reflects a growing recognition that emotional processes drive not only psychological but also sociocultural dynamics in visitor experiences.

One widely recognized perspective focuses on the psychological dimension of emotions, highlighting both cognitive and affective factors that guide tourists’ choices and behaviors (Yang et al., 2021; Yang et al., 2020). Here, “cognitive” encompasses awareness, interpretation, and thought processes, while “affective” refers to immediate feelings and mood states. For instance, Yang et al. explored how sensory elements at a destination can foster visitor loyalty by uncovering internal mechanisms that shape tourism experiences (Yang et al., 2021). Another study by Yang et al. investigated self-congruity as an intermediary between destination attributes and tourist behavioral intentions, while also noting that emotional experiences can moderate these relationships (Yang et al., 2020). This underscores how personal identity intersects with emotional responses, potentially strengthening or weakening tourists’ commitment.

From a sociological standpoint, emotional experience is examined within broader social and cultural frameworks. Medina et al. studied tourist motivations in historical World Heritage cities, emphasizing that cultural identity often intertwines with emotional bonding and can strongly influence visitor satisfaction (Medina-Viruel et al., 2019). Such findings highlight how deeper cultural resonance can intensify affective responses, affecting behavioral outcomes like loyalty (Yang et al., 2021) and subjective well-being (Al-okaily et al., 2023). This connection emphasizes the importance of cultural nuance in sustainable tourism management and the refinement of visitor experiences.

Recent studies increasingly employ either cross-sectional or longitudinal methods to explore how emotions function in tourism. Cross-sectional designs capture a single “snapshot” of emotional states, revealing how emotions impact specific tourism contexts such as food tourism (Lai et al., 2021), cruise experiences (Lyu et al., 2018), or sports events (Joo et al., 2019). Lai et al. (2021) demonstrated that emotional value—derived from online reviews—significantly determines visitor satisfaction with culinary experiences. Similarly, Lyu et al. (2018) built a psychological model illustrating how emotional, relational, and cognitive engagement in cruise contexts can enhance tourist well-being. These investigations often categorize emotions into positive and negative dimensions, yet such valence-based approaches risk oversimplifying a more nuanced affective landscape.

Longitudinal research, on the other hand, tracks the evolution of emotions from generalized affective responses toward more specific emotional experiences over time, including nostalgia (Zou et al., 2021), awe (Su et al., 2020), and profound emotional bonds shaped during travel (Jiang et al., 2022; Zhou et al., 2023). Zou et al. (2021) examined how overseas Chinese travelers cultivate emotional ties to ancestral hometowns, contributing to diaspora tourism discussions. Su et al. (2020) focusing on visitor experiences at Meizhou Island, identified awe as a mediating factor between engagement and loyalty, reinforcing how repeated or prolonged encounters with a destination can deepen emotional attachment. Such repeated engagements may build emotional memory and anticipation, thus shaping ongoing tourism experiences.

In summary, disciplines such as human geography and tourism sociology acknowledge that emotions significantly shape travel experiences. Nevertheless, many studies default to broad positive–negative distinctions, offering limited insight into the cognitive and multi-dimensional aspects of emotions. Future research could use more intricate measures—such as pleasure, arousal, and immersion—and incorporate variables like attention or personal meaning to capture subtle variations in travelers’ emotional journeys. Integrating richer, quantitative data on emotional complexity would thus shed new light on how emotions act as a key driver in shaping tourism experiences.

### 2.3 SOR model and mediating effect theory

The Stimulus-Organism-Reaction (SOR) model originated from the behaviorist stimulus–response framework and was later refined by Mehrabian et al., establishing its role in explaining how environmental factors influence human psychology and behavior (Mehrabian and Russell, 1974). The fundamental principle of this model asserts that external stimuli do not directly trigger behavioral responses; instead, they are processed through an individual’s internal state and psychological mechanisms. This means that a person’s reaction to external conditions is not solely dependent on the stimulus itself but is also shaped by factors such as personal traits, attitudes, emotions, and expectations. The SOR framework, therefore, consists of three key phases—stimulus, organism, and response—emphasizing the critical role of internal mediation between external triggers and behavioral outcomes (Mehrabian and Russell, 1974; Hayn-Leichsenring, 2017).

Recently, the SOR model has been extensively applied in fields such as consumer tourism management, consumer behavior analysis, and consumer psychology, due to its ability to comprehensively assess the relationship between external influences and consumer responses. The scope of SOR-based studies has expanded beyond traditional tourism landscapes, incorporating elements such as visual gamification as independent variables and tourists’ behavioral responses as mediators influencing final outcomes.

For instance, Kumar et al. (2021) utilized SOR theory to conceptualize how an application’s aesthetic design influences pleasure, arousal, intention to revisit, and word-of-mouth recommendations. Wang et al. (2023) integrated SOR theory with flow theory to examine how the presentation of famous artworks in VR impacts tourists’ engagement and technology adoption. Similarly, Thomas and Baral explored the role of gamified learning environments in shaping cognitive, behavioral, and emotional engagement within educational contexts (Thomas and Baral, 2023).

While these studies highlight how different art-related environments influence psychological perceptions and behavioral patterns, there remains a research gap in applying SOR theory to understand how art spaces affect visitor satisfaction. There is also a lack of studies investigating how emotional regulation strategies can enhance art tourism experiences. Addressing these gaps, applying the SOR framework to analyze tourist satisfaction provides a structured approach to examining how cognitive, emotional, and behavioral reactions evolve in artistic environments, offering valuable insights for art tourism destination planning and visitor experience enhancement.

A mediating effect occurs when the relationship between two variables is not a direct causal link but is instead influenced by one or more intermediary variables that act as indirect channels of impact. In short, Mis a function ofX, Yis a function of M, and the independent variable(X)affects the dependent variable(Y) through a certain variable(M), and is called an intermediary variable. When a model includes a single mediating variable, the mediating effect is equivalent to the indirect effect. However, in cases where multiple mediating variables exist, the overall mediating effect does not necessarily equal the sum of the individual indirect effects (Yan et al., 2023). To illustrate this concept, consider a basic three-variable model. Assuming all variables have been centralized, the mediation relationship can be mathematically represented by the following equations:


(1) $$Y=\mathrm{cX}+\varepsilon_{1}$$

(2) $$M=\mathrm{aX}+\varepsilon_{2}$$

(3) $$Y=c\prime X+\mathrm{bM}+\varepsilon_{3}$$

The coefficient of Equation 1 c is the total effect of the independent variable(X)on the dependent variable(Y); the coefficient of Equation 2 a is the effect of the independent variable(X)on the mediating variable(M); the coefficient of Equation 3 bis the effect of the mediating variable(M)on the dependent variable(Y) after controlling the impact of the independent variable(X); the coefficient c′is the direct effect of the independent variable(X) on the dependent variable(Y) after controlling the influence of the mediating variable(M); $\varepsilon_{1}\sim \varepsilon_{3}$ is the regression residual.

The mediation effect model is a statistical approach used to analyze the influence of mediating variables in explaining the relationship between an independent variable and a dependent variable. Through path analysis and related methods, this model quantifies the size and significance of the mediation effect, helping to clarify how and why an independent variable impacts an outcome variable (Cuny et al., 2020). The core principle of the mediation effect model is to statistically assess whether the influence of the independent variable on the dependent variable occurs directly or needs to be transmitted through a mediating variable. This analytical method not only provides insight into underlying mechanisms but also facilitates the integration of existing theories and empirical research. By quantifying relationships between variables, researchers can systematically explore the extent to which independent variables influence dependent variables, making the model highly valuable in both theoretical and practical contexts.

However, despite its advantages, the application of the mediation effect model has limitations. Since the selection of mediating variables is often based on assumptions derived from prior research, subjectivity plays a role in determining which mediating variables are included in the analysis. As a result, the validity and reliability of findings may be constrained by the theoretical foundation on which the model is built.

In this study, we integrate the SOR model with mediation effect theory to strengthen the logical structure and theoretical persuasiveness of our research framework. Given that the mediation effect model is widely used to analyze decision-making processes and individual behavioral responses, it has increasingly been applied in tourism research to explain visitor experiences and behavioral intentions. Specifically, recent studies have employed mediation models to explore variables such as comfort (Yan et al., 2023), satisfaction (Wei et al., 2023), loyalty (Yang et al., 2021; Patwardhan et al., 2020), and environmentally responsible behavior (Weng et al., 2022). Table 2 presents a selection of representative studies from recent years that highlight these applications.

**Table 2:** Representative literature on mediating effects in tourism and art research.

Author	Published time	Research contribution
Yan et al. (2019)	2023	Analyzing the influence of different landscape features on emotional responses and thermal comfort, revealing that emotions act as a mediator in the relationship between landscape elements and perceived thermal comfort.
Wei et al. (2023)	2023	Investigating the connection between gamification, travel fatigue, and tourist satisfaction, identifying that motivation fatigue serves as a mediator in the relationship between gamification and travel satisfaction.
Patwardhan et al. (2020)	2020	Analyzing tourists’ emotional bonds with destinations in India, focusing on emotional solidarity and assessing how perceived safety influences destination loyalty
Weng et al. (2022)	2022	Identified that environmental attitude partially mediates the relationship between entertainment experience and environmentally responsible behavior.
Wang et al. (2023)	2022	Investigate how VR engagement influences behavioral intentions and offer innovative strategies for destination marketing.
Kumar and Shah (2021)	2021	Examining how an app’s aesthetic design mediates emotional responses, enhancing the understanding of its impact on users’ intention to continue using the app.
Lee and Kim (2022)	2022	Analyzing the mediating roles of perceived novelty and typicality in the relationship between color design and advertising attitudes, offering strategic recommendations for marketers on effectively integrating these elements to capture consumer interest.
Quach et al. (2022)	2022	Develop a conceptual framework illustrating the influence of artistic infusion on brand evaluation, while examining the mediating and moderating roles of factors such as value expression, social adaptation, and self-untrue experience.
Liu et al. (2023)	2022	Investigates the mediating and moderating effects in the relationship between souvenir symmetrical design and tourists’ aesthetic pleasure, offering insights for industry professionals on leveraging souvenir design to enhance the tourist experience.

Recent studies on landscape and artistic environments frequently integrate mediation effect theory, as shown in Table 2. Unlike the SOR model, which examines the connection between external stimuli and behavioral responses, mediation effect theory focuses primarily on cognitive processing, often neglecting underlying mechanisms and moderating influences. By incorporating the SOR framework, this study offers a more comprehensive perspective on how individuals interpret and react to environmental stimuli, providing a deeper understanding of the factors shaping satisfaction with artistic landscapes.

Although previous research on landscape satisfaction, emotional experience, and mediation effects has contributed to urban planning, tourism management, and consumer behavior studies, several gaps remain. Many prior studies on landscape satisfaction have relied on broad analyses of residents’ and tourists’ perceptions of natural environments, frequently employing SWOT and IPA methodologies. However, there has been little exploration of the mechanisms influencing tourist satisfaction in public art spaces and artistic tourism destinations from a psychological perspective. Furthermore, while existing research on emotional experience has examined how emotions shape tourist behavior, it has often overlooked the combined influence of emotion and cognition. This study introduces cultural identity as a moderating factor, aiming to analyze how socio-cultural backgrounds shape the impact of visual stimuli on tourists’ perception and satisfaction in art spaces.

Much of the literature on emotions in tourism has primarily classified emotions as either positive or negative, with limited attention given to specific emotional responses in unique tourism contexts. Since aesthetic appreciation is a fundamental component of art tourism, this study integrates aesthetic experience into the analysis of satisfaction in art districts. Additionally, mediation effect models often fail to account for key antecedent variables and moderating factors, such as the natural environment, interpersonal interactions, and socio-cultural influences. By combining the SOR model with mediation effect analysis, this study provides a more holistic approach to understanding the psychological processes of tourists, ultimately offering strategic recommendations for managing and developing art tourism destinations. Through this integration, the research aims to bridge existing gaps and present a more refined perspective on how visitors experience and evaluate artistic environments.

## 3 Assumptions

Across diverse tourism scenarios, numerous studies have consistently shown that perceived tourism product quality strongly correlates with tourist satisfaction, reaffirming the notion that the quality of destination products is a key driving factor (Xie et al., 2023; González-Rodríguez et al., 2019).

More recently, scholars have underscored the importance of visual aesthetic quality in understanding consumer behavioral intentions. Kumar et al. investigated how an application’s aesthetic form influences both revisit intention and word-of-mouth, ultimately proposing a conceptual model to clarify this relationship (Kumar et al., 2021). Zhang and Xu (2020) examined how aesthetic quality shapes loyalty in the context of nature tourism. Meanwhile, El-Adly analyzed hotel tourist loyalty mechanisms, concluding that customer satisfaction exerts a direct positive impact on loyalty (El-Adly, 2019).

Building on these insights, it is plausible that visual quality exerts an influence on tourist satisfaction akin to its effect on loyalty. Hence, we propose:

*H1:* The visual quality of art districts positively influences tourist satisfaction.

As the primary external stimulus perceived by individuals, visual elements are processed through cognitive functions such as recognition, comparison, and interpretation, eventually shaping aesthetic and emotional responses (Stein and Ohler, 2017). When visitors experience the visual appeal of an art district, they engage in an aesthetic appreciation that is distinct from everyday perception (Leder et al., 2004; Mastandrea et al., 2019). Research has shown that visual attributes of buildings and landscapes—such as architectural form, color, spatial openness, and natural elements—significantly influence emotional responses such as arousal and pleasure (St-Jean et al., 2022). Sevenant and Antrop further highlighted that high-quality landscapes evoke positive emotions, including happiness and security, whereas low-quality landscapes can trigger negative emotions such as stress and fear (Sevenant and Antrop, 2010). Additionally, Kumar and Shah examined the impact of aesthetic web design on consumer emotions, demonstrating that aesthetically pleasing interfaces enhance positive emotional experiences (Kumar and Shah, 2021).

Applying these principles to tourist destinations, it is likely that higher perceived visual quality in an art district enhances tourists’ aesthetic experiences, while lower visual quality diminishes it. Based on this, we propose the following hypothesis:

*H2:* The visual quality of art districts positively influences tourists' aesthetic experience.

The SOR (Stimulus-Organism-Response) theory has been extensively applied in tourism management (Kim et al., 2020), consumer behavior (Domínguez-Quintero et al., 2019), and consumer psychology (González-Rodríguez et al., 2019) to explore how external stimuli influence internal psychological states, which in turn shape behavioral responses. In the context of public art spaces, visual quality serves as an external stimulus, aesthetic experience represents the internal emotional and cognitive response induced by the visual environment, and tourist satisfaction reflects the outcome of this experience.

The visual quality of an art district acts as a sensory stimulant, fostering art appreciation and evoking aesthetic experiences (Tang et al., 2020). Numerous studies have recognized the importance of aesthetic experience in shaping tourist feedback and behavioral intentions (Quadri-Felitti and Fiore, 2016). Mehmetoglu & Engen examined music festivals and museum visits, concluding that aesthetic experience was the only factor in tourist experiences that had a direct positive impact on satisfaction (Mehmetoglu and Engen, 2011). Similarly, Song et al. applied experience economy theory to investigate the relationship between tourism experience and satisfaction, finding that aesthetic experience enhances emotional value, which in turn affects satisfaction levels (Song et al., 2015).

From these findings, we can infer that when the visual environment of a destination effectively induces aesthetic experiences, tourists are more likely to report higher satisfaction. Therefore, we propose the following hypotheses:

*H3:* Aesthetic experience positively influences tourist satisfaction.

*H4:* Aesthetic experience mediates the relationship between visual quality and tourist satisfaction.

## 4 Methodology

### 4.1 Measurement framework

The visual quality of landscapes is conceptualized based on a comprehensive evaluation of visual characteristics across various theoretical perspectives. However, there is no universal agreement on the selection of evaluation indicators for different types of landscapes (Tveit et al., 2006; Kalivoda et al., 2014). To address this gap, this study develops a measurement scale for the visual quality of art districts by drawing from a broad literature base on tourist destination aesthetics and visual landscape assessment frameworks. Since art districts possess aesthetic value derived from their formal attributes while simultaneously conveying cultural significance, this study classifies visual quality into three dimensions: aesthetic quality, cultural quality, and environmental quality. The scale is adapted from previous works, including those of Kirillova et al. (2014), Tveit et al. (2006), and Kaplan and Kaplan (1989), to assess the overall visual appeal of art districts. While these measurement tools have been validated in past studies, they were primarily applied to natural landscape environments, and their applicability to urban art venues requires further verification.

To ensure methodological rigor, this study follows a structured data processing approach. First, an exploratory factor analysis (EFA) is conducted to refine the dimensions of visual quality by identifying underlying factor structures. The factor extraction process adheres to the eigenvalue-greater-than-one criterion. Second, confirmatory factor analysis (CFA) is employed to test the reliability and validity of the refined measurement model, using model fit indices such as CFI, TLI, RMSEA, and SRMR to confirm goodness-of-fit. Third, structural equation modeling (SEM) is applied to examine the hypothesized relationships among visual quality, aesthetic experience, cultural identity, and tourist satisfaction, ensuring robust hypothesis testing.

In addition to visual quality, aesthetic experience is defined as the cognitive judgments and emotional responses elicited in tourists upon exposure to a destination’s visual stimuli. This study employs Leder et al. (2004) approach to measure aesthetic experience, which conceptualizes aesthetic experience through a multi-stage process. Specifically, the model consists of five key dimensions: (1) perceptual analysis, where individuals recognize basic structural elements of the visual stimulus; (2) implicit memory integration, which links prior experiences and learned preferences to new stimuli; (3) explicit classification, where individuals categorize and interpret the artwork based on cultural or stylistic knowledge; (4) cognitive mastering, involving a deeper understanding of the meaning and artistic composition; and (5) emotional evaluation, where an individual experiences an affective response such as pleasure, awe, or curiosity. This structured approach ensures a comprehensive assessment of how aesthetic stimuli influence visitor perception and engagement. Cultural identity, another key variable, is assessed using the three-item scale from Yang et al. (2022) research. Furthermore, tourist satisfaction, a critical concept in tourism management and destination planning, reflects the extent to which a visitor’s expectations align with their actual experience. This study adopts Yoon and Uysal (2005) single-item measurement to evaluate satisfaction.

Additionally, the survey collects demographic information, including gender, age, education level, and occupation. All questionnaire items are rated on a five-point Likert scale, where 1 represents strong disagreement and 5 indicates strong agreement.

### 4.2 Data description

Between August and September 2023, this study employed a combination of convenience sampling and snowball sampling to gather data from tourists visiting selected art tourism destinations. Participants were initially invited to complete a survey on-site, and afterward, they were asked to distribute an online questionnaire to acquaintances who had also experienced art districts, enabling snowball sampling.

In accordance with previous research, a larger sample size enhances the stability of statistical analysis and improves the practicality of indicators when using structural equation modeling (SEM) (Wolf et al., 2013; Iacobucci, 2010). To ensure a sufficient number of valid responses, a total of 700 questionnaires were distributed. After removing invalid responses, 603 valid questionnaires were retained, yielding an effective response rate of 86.1%, which meets the standard requirements for sample size adequacy in empirical research.

Additionally, to refine the structure of visual quality, a pretest involving 150 questionnaires was conducted using exploratory factor analysis (EFA). The remaining 453 responses were utilized for the main empirical analysis in this study.

## 5 Result analysis

This study employs mean and frequency analysis to summarize the demographic characteristics and travel profiles of the respondents. To assess the dimensional structure of the visual quality scale, exploratory factor analysis (EFA) was conducted.

Subsequently, structural equation modeling (SEM) was performed using Mplus version 8.0 to examine the relationships among the four constructs proposed in the conceptual framework. The SEM analysis follows a two-stage approach: 1. Confirmatory factor analysis (CFA) to validate the factor structure and assess the model fit. 2. Structural model testing to analyze the relationships between visual quality, aesthetic experience, cultural identity, and tourist satisfaction. 3. Multiple regression analysis to evaluate the moderating effect within the proposed framework.

### 5.1 Demographics

**Figure 1:**
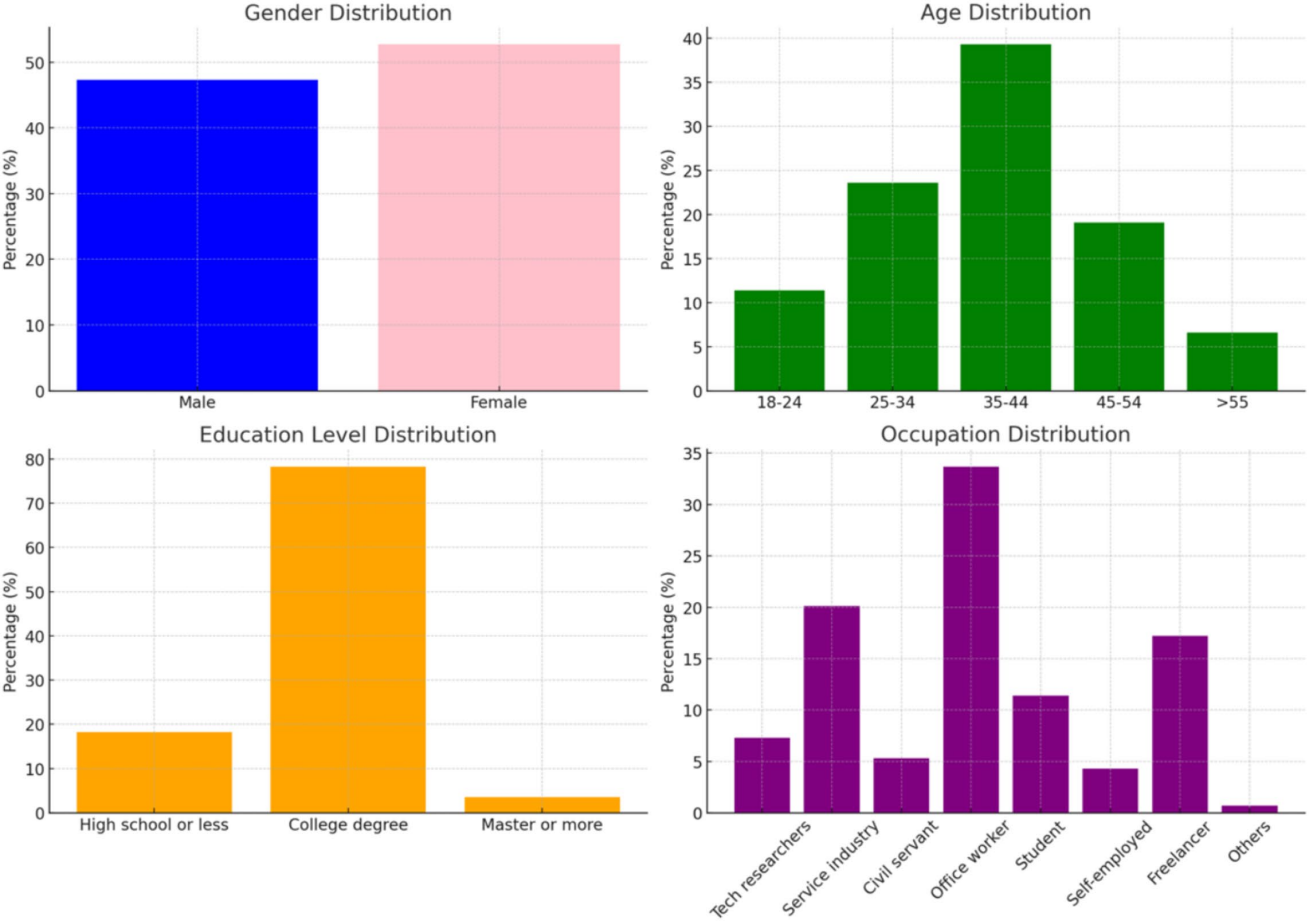
Demographic characteristics of respondents in art tourism study.

The demographic composition of the participants includes 47.3% men and 52.7% women. In terms of age distribution, the majority (82%) fall within the 25 to 54 age range. Regarding educational background, 81.8% of respondents hold at least a college degree. Occupationally, most participants are employed in the service industry or work as office employees, collectively making up 53.8% of the total sample, as illustrated in Figure 1.

### 5.2 EFA

Exploratory Factor Analysis (EFA) was conducted using SPSS 23.0, applying Principal Component Analysis (PCA) with Varimax rotation on the pre-survey sample (*n* = 150) to refine the dimensions of visual quality. The analysis identified a three-factor structure, using eigenvalues greater than 1 as the criterion for factor extraction. The final model retained three distinct factors—aesthetic quality, cultural quality, and environmental quality—which together explained 69.537% of the variance in visual quality. The first factor represents the aesthetic attributes that shape tourists’ visual experience, the second factor reflects tourists’ recognition and acceptance of cultural characteristics, and the third factor captures the influence of environmental attributes on tourists’ overall experience.

**Figure 2:**
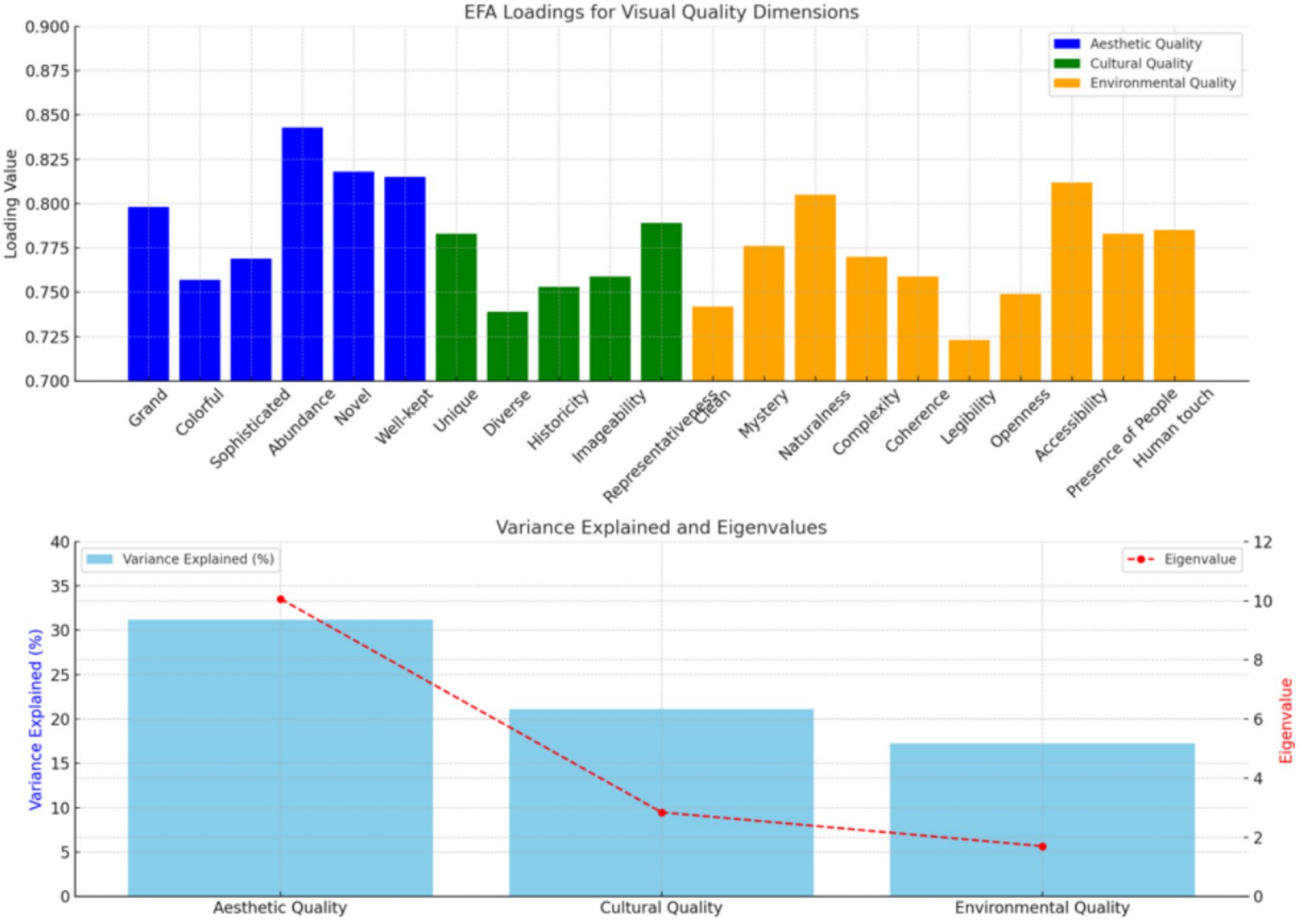
EFA results for visual quality.

As shown in Figure 2, all factor loadings exceed 0.7, demonstrating strong explanatory power, leading to the retention of all items. The KMO test yielded a coefficient of 0.942, surpassing the 0.7 threshold, confirming the dataset’s suitability for factor analysis (Tabachnick and Fidell, 2007). Additionally, Bartlett’s sphericity test produced an approximate chi-square value of 2271.289 with 210 degrees of freedom, and a significance level of <0.001, indicating that the dataset adheres to a multivariate normal distribution and is statistically significant (Kaiser, 1960).

### 5.3 Structural model evaluation

The model fit indices indicate that the model aligns well with the data, as evidenced by χ^2^ = 362.155, χ^2^/df = 2.236, RMSEA = 0.052 (< 0.08), SRMR = 0.092 (> 0.9), CFI = 0.968 (> 0.9), and TLI = 0.962 (> 0.9). These results confirm that the structural model meets the recommended fit criteria.

The estimated path coefficients further support the research hypotheses. Visual quality exhibits a significant positive effect on tourist satisfaction (*γ* = 0.445, *p* < 0.001, CI = 0.330–0.563), and aesthetic experience also significantly enhances tourist satisfaction (*γ* = 0.313, *p* < 0.001, CI = 0.194–0.430), thereby supporting H1 and H3. Additionally, visual quality demonstrates a significant positive impact on aesthetic experience (*γ* = 0.572, *p* < 0.001, CI = 0.488–0.643), validating H2.

To assess the mediating effect of aesthetic experience between visual quality and tourist satisfaction, a bootstrap method with 5,000 resamples was conducted. Mediation is confirmed if the 95% bias-corrected confidence interval (BC CI) does not include zero. The results show that *γ* = 0.179, *p* < 0.001, CI = 0.112–0.255, verifying H4 and indicating that aesthetic experience plays a mediating role in the relationship between visual quality and tourist satisfaction.

## 6 Discussion and conclusion

Analyzing how visual elements in artistic environments shape visitor satisfaction is fundamental for improving the planning and management of creative tourism destinations. Despite its significance, there has been limited research on the interplay between public art and tourist behavioral outcomes from an emotional experience perspective (Tang et al., 2020). This study introduces a structured conceptual framework, synthesizing previous research and cognitive psychological principles under the SOR model. By establishing a mediation model, this study clarifies the relationship between visual quality, aesthetic experience, cultural identity, and visitor satisfaction, offering valuable insights for the management and development of art spaces.

1. Understanding Visual Quality’s Role in Tourist Satisfaction: This study confirms that higher visual quality contributes positively to both aesthetic experience and overall satisfaction. The findings align with previous research emphasizing that visual design significantly influences cognitive perception, emotions, and visitor responses (St-Jean et al., 2022). A well-balanced aesthetic composition enhances emotional appeal and fosters positive affective engagement (Motoyama and Hanyu, 2014; Tang and Long, 2019).
2. Psychological Mechanisms of Aesthetic Experience: This study further confirms the mediating role of aesthetic experience in the relationship between visual quality and visitor satisfaction. The results reveal the psychological mechanisms through which the visual appeal of art districts influences tourists’ perceptions by evoking pleasurable and immersive aesthetic emotions (Kumar et al., 2017). Drawing upon cognitive psychology and environmental aesthetics theories, we suggest that visual stimuli are processed through both cognitive and affective pathways, influencing individuals’ emotional engagement and overall evaluation of a space.
3. Role of Cultural Identity in Aesthetic Perception: Cultural identity enhances aesthetic perception and visitor engagement. Exposure to cultural elements fosters deeper appreciation, interpretation, and interactive participation, strengthening aesthetic enjoyment and visitor satisfaction (Medina-Viruel et al., 2019; Prayag et al., 2020). Incorporating intangible heritage elements (e.g., mythology, poetry, historical symbolism) enriches visitor experiences by evoking reminiscence and imagination (Zhang and Brown, 2023).
4. Interactive and Co-Creation Strategies for Art Spaces: Future art districts could integrate co-creation activities where visitors engage directly with artists. Allowing visitors to modify form, color, and texture fosters meaningful exchanges between designers and visitors, enriching the artistic experience beyond passive observation.
5. Recommendations for Future Art Space Management: Art spaces should prioritize visual quality that aligns with visitors’ aesthetic responses and cultural connections. Strengthening emotional engagement between creators and visitors can enhance the interpretive depth of artistic environments. Visual elements should contribute to immersive and fulfilling visitor experiences rather than merely serving decorative purposes.

In conclusion, this study provides a structured examination of how visual quality influences visitor satisfaction in artistic environments, highlighting the mediating role of aesthetic experience and the moderating role of cultural identity. By offering insights into the psychological mechanisms underlying these relationships, the findings contribute to both theoretical and practical discussions on the optimization of visual experiences in creative tourism destinations. Strengthening visitor engagement through well-balanced visual design and interactive opportunities will be essential for the sustainable management and future development of art districts.

## Data Availability

The original contributions presented in the study are included in the article/supplementary material, further inquiries can be directed to the corresponding author/s.
